# Impact of Host and Management Factors on Calf Morbidity and Mortality Rates in Smallholder Dairy Farms in Central Ethiopia: A Prospective Cohort Study

**DOI:** 10.1155/vmi/8463332

**Published:** 2025-07-07

**Authors:** Biruk Alemu, Gizachew Hailegebreal, Rahmeto Abebe

**Affiliations:** ^1^Afar Field Office, CARE International, Semera, Ethiopia; ^2^Faculty of Veterinary Medicine, Hawassa University, P.O. Box 05, Hawassa, Ethiopia

**Keywords:** calf, central region, dairy farm, incidence, morbidity, mortality

## Abstract

The dairy sector in Ethiopia is vital for the agricultural economy and smallholder farmers; however, calf morbidity and mortality present significant challenges. A prospective longitudinal study conducted tracked 204 newborn calves across 120 farms in central Ethiopia to estimate morbidity and mortality rates, identify causes, and assess risk factors. The calves were monitored every 15 days until they reached 6 months of age. Data analysis utilized Kaplan–Meier survival analysis and Cox proportional hazard regression. The study found a morbidity rate of 13.4 and a mortality rate of 4 cases per 100 calf-months at risk. Diarrhea was the most commonly diagnosed condition, accounting for 50.5% of morbidity and 64.5% of mortality. Key risk factors for morbidity included calving assistance (HR = 1.93), floor structure (HR = 2.88), calf sex (HR = 1.86), late colostrum intake (HR = 1.7), weaning age (HR = 0.47), dam breed (HR = 0.21), and calf age (HR = 0.23). Risk factors for mortality included farm location (HR = 0.25), calving assistance (HR = 7.7), birth site (HR = 27.3), floor structure (HR = 9.18), late colostrum intake (HR = 7.68), weaning age (HR = 0.03), and calf age (HR = 0.15). The observed morbidity and mortality rates exceed acceptable levels, jeopardizing calf health and dairy sector growth. Enhancing management practices—such as timely colostrum provision, early disease detection and treatment, and farmer education—is crucial to mitigate these rates. Further research is needed to pinpoint specific causes of calf morbidity and mortality in the study areas.

## 1. Introduction

Ethiopia has the largest dairy cattle population in Africa, primarily managed through traditional husbandry, alongside a few semiintensive to intensive commercial farms in urban and periurban areas. The dairy sector is crucial to the agricultural economy, supporting smallholder farmers and enhancing food security and economic growth [1, 2]. However, the sector faces several significant challenges, including inadequate feed quality and quantity, limited land for improved forage, insufficient veterinary services, diseases, lack of improved breeds, poor artificial insemination (AI), inadequate animal management, and a lack of market-oriented production [3]. Calf morbidity and mortality are major issues affecting dairy cattle production in Ethiopia and worldwide [4, 5].

High rates of calf morbidity and mortality significantly impact the economic viability of the dairy industry, as calf health is crucial for the productivity of future herds. Factors contributing to these issues include inadequate nutrition and poor management practices among smallholder dairy farms [5–7]. The main losses faced by the dairy industry include calf deaths, increased healthcare costs, and a decrease in lifetime productivity. Additionally, calf mortality leads to the loss of valuable genetic materials for herds [8, 9]. Research indicates that calf morbidity and mortality problems persistently trouble dairy farmers, particularly in the first 6 months of a calf's life, highlighting the need for effective health control and prevention programs to address and mitigate these challenges [10–12]. Worldwide, diarrhea and pneumonia account for the majority of morbidity and mortality in calves [13].

Calf morbidity and mortality are major challenges for dairy production and farmers' livelihoods in Ethiopia. Several studies have documented calf morbidity rates ranging from 22% to 66.8% and mortality rates between 0.9% and 37% across various regions [12, 14]. Notably, most reported mortality rates exceed the economically acceptable threshold of 3%–5%, which is considered the minimum standard for western production systems [15]. This highlights the need for further research and the development of evidence-based strategies to mitigate calf mortality, especially on emerging commercial dairy farms in urban and periurban areas. However, much of the existing data on calf morbidity and mortality come from cross-sectional studies, which provide limited insight into the problem and its risk factors. This study adopts a prospective longitudinal design, offering advantages over cross-sectional approaches, particularly in animal disease research. Longitudinal studies track the same subjects over time, facilitating the observation of disease progression and the identification of cause-and-effect relationships. They yield more detailed data, allowing researchers to control for various background characteristics and reduce confounding variables. Moreover, longitudinal studies can capture changes in the health status, behavior, or environmental factors that may not be apparent in the single snapshot provided by cross-sectional studies [16].

In Ethiopia, few longitudinal studies have examined the incidence of morbidity and mortality in calves and their associated risk factors [10–12, 17]. These studies indicate high rates of calf morbidity and mortality, with one reporting a morbidity rate of 55 per 100 calves over six months and a mortality rate of 14 per 100 calves [12]. Diarrhea and pneumonia are the most prevalent diseases among calves, contributing significantly to morbidity and mortality. Identified risk factors include dam parity, pen cleaning practices, floor type, and the timing of colostrum feeding [12]. However, these studies are limited to specific urban areas in the country.

There is a lack of sufficient reports on calf morbidity and mortality rates in Ethiopia's central regional state, despite the presence of numerous smallholder dairy farms. Continued research and development in calf health and management are crucial for creating effective interventions. To enhance calf growth sustainability on small-scale dairy farms, prospective cohort studies are necessary for thorough problem investigation. This research aims to address specific issues related to calf morbidity and mortality, ultimately informing evidence-based interventions that can improve calf health, strengthen dairy production systems, and benefit smallholder farmers and the wider dairy industry. Thus, the present study seeks to estimate calf morbidity and mortality rates, identify major causes, and recognize associated risk factors in urban and periurban smallholder dairy farms across selected districts in the central regional state of Ethiopia.

## 2. Materials and Methods

### 2.1. Study Area

**Figure 1:**
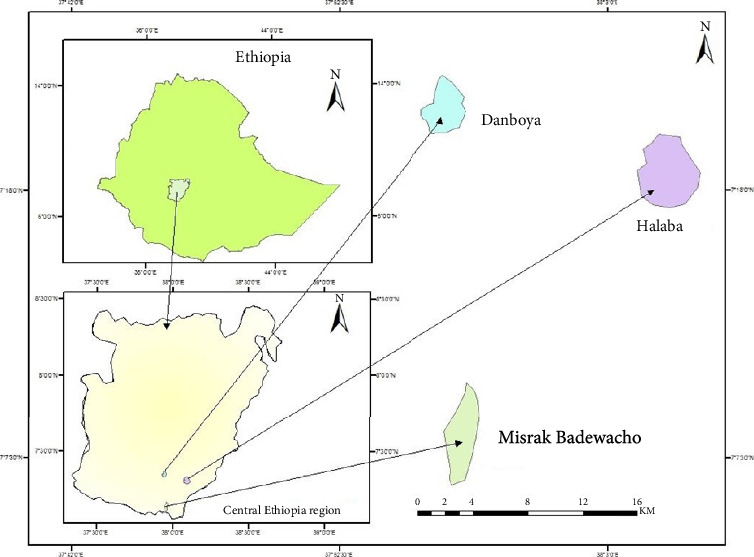
Map of Ethiopia showing the central Ethiopian region and the three selected districts.

This study took place from December 2022 to June 2023 on urban and periurban dairy farms in the central Ethiopia region, specifically in the Misrak Badewacho, Danboya, and Halaba districts (Figure 1). Misrak Badewacho district is located in the Hadiya zone of the central Ethiopian region, between 07° 03′ 20″ N to 07° 16′ 08″ N of latitudes and 037° 53′ 02″ E to 038° 06′ 02″ E of longitude. Its elevation ranges from 1650–2050 m above the sea level. The annual rainfall ranges between 800 and 1500 mm, and the annual temperature ranges between 18.5°C and 22.5°C. The primary purpose of raising cattle in the district is for milk production. The district heavily relies on natural mating for breeding due to the delayed AI service availability, distance from AI stations, and technician shortages for farmers (Amanuel and Eskindir, 2023, unpublished).

The Danboya district presents significant potential for dairy cattle farming, located at 6° 23′ 30″ N latitude and 36° 7′ 23″ E longitude within the Kambata zone. This area is pivotal to the Ethiopian government's goal of quadrupling milk production by 2031 by boosting dairy cow productivity. Situated about 285 km southwest of Addis Ababa, Danboya spans an altitude of 1501 to 2500 m above the sea level, with an average annual rainfall of 1200 to 1800 mm and temperatures between 19°C and 29°C. Covering 151.83 square kilometers, the district offers ample opportunity for dairy farming initiatives [18].

The Halaba district is situated in the Halaba zone of the central Ethiopian region, between latitudes 7°10′N and 7°42′N and longitudes 38°00′E and 38°25′E. It is located approximately 315 km south of Addis Ababa. The altitude in the area ranges from 1554 to 2149 m above the sea level. The mean annual rainfall varies between 544 and 1271 mm. The mean monthly minimum and maximum temperatures are 12.8°C and 26.8°C, respectively. The district boasts favorable agro-ecological conditions, ample land for pasture, and a rising demand for dairy products within the district. Known for its suitable climate, well-connected roadways, and availability of improved dairy cattle breeds, the district provides essential factors for successful dairy farming. Consequently, dairy production is rapidly expanding in the area, with numerous households relying on it for both income and consumption (Halaba Zone Finance and Economic Development Office, 2019; unpublished).

Commonly grown crops in the region include teff, wheat, barley, maize, enset (false banana), and various vegetables. These crops and their residues are used as feed sources for the urban and periurban dairy farms in the area.

### 2.2. Study Population

The study targeted dairy calves (≤ 6 months old) reared on smallholder farms across three districts. These farms are predominantly family-operated and specialized in milk production from a limited number of cattle. In all three districts, cattle are the dominant livestock species followed by sheep and goats. District-wide cattle populations range from approximately 50,000 to 81,653 heads. In Misrak Badewacho, cattle are mostly indigenous breeds with a small proportion of exotic or crossbred animals. Conversely, in Halaba and Danboya districts—particularly in urban and periurban areas—crossbred cattle predominate. Our sample specifically focused on farms located in the central towns and surrounding villages of the three districts. Registered smallholder dairy farms in the aforementioned locations numbered approximately 71 in Misrak Badewacho, 81 in Halaba, and 74 in Danboya, forming the basis of our study population.

Breeding strategies vary across districts, but farmers generally maintain diverse herd sizes to ensure a consistent milk supply year-round. In Halaba, farmers often utilize both traditional and improved breeding methods, increasingly using AI to enhance milk yield and quality. They engage in crossbreeding with higher-yield breeds such as Holstein or Jersey through AI programs. In contrast, traditional breeding methods prevail in Misrak Badewacho district, where AI access is limited, leading farmers to rely on locally adapted breeds suited to their climate. Danboya district, however, adopts a more progressive approach with better AI access, often crossbreeding local cattle with high-yield breeds such as Holstein-Friesian. The predominant cattle in Danboya are crossbreeds, which adapt well to the environment but lack the resilience of local breeds.

Management and feeding practices vary across the districts, but all districts prioritize optimizing milk production and herd health. Feeding practices include grain and hay supplementation, with distinction between districts. In Misrak Badewacho, grazing and crop residues are the primary feed, complemented by locally available supplements. In Halaba and Danboya, grazing and crop residues persist, but farmers place greater emphasis on grain supplements.

Urban and periurban dairy production systems in the study districts show significant differences in management practices, which directly affect calf morbidity and mortality rates. Urban farms in these districts typically operate under semi-intensive systems, where calves are housed in barns and receive supplements of grain and hay. These farms usually have smaller herd sizes due to limited land availability, but they implement relatively better biosecurity measures and invest more direct labor per animal. Furthermore, the veterinary services are better in the urban farms compared with the periurban areas.

In contrast, periurban farms primarily utilize extensive production systems, characterized by grazing and the use of crop residues, along with supplemental feeding. These farms have larger herd sizes because of the availability of more land and tend to rely more on family labor.

Dairy farms are classified by herd size—following Kidane et al. [19]—into smallholder (1–5 cows), medium (6–30 cows), and large (> 30 cows). Calves included in the study were both local and crossbred, of either sex, aged from birth to 6 months, and raised on smallholder farms within the selected urban and periurban districts. Excluded were stillborn calves, those presenting congenital deformities or wasting diseases, and any calves born outside the defined study areas.

### 2.3. Study Design

An observational prospective longitudinal study was conducted to monitor newborn calves over 6 months and survey cattle owners about their management practices. This design was preferred over cross-sectional studies for its capacity to establish causal relationships. Calves were the primary sampling units and were individually monitored throughout the study. The Halaba, Danboya, and Misrak Badewacho districts were intentionally selected due to their higher potential for dairy production in central Ethiopia.

Data from the district agriculture offices provided the sampling frame for the study, which included 83, 74, and 71 registered smallholder dairy farms in Halaba, Danboya, and Misrak Badewacho, respectively. A simple random sampling technique, using a lottery method, was employed to select farms until the desired sample size was achieved. If a selected farmer declined to participate or did not have a calf or pregnant cows with a due calving date during the follow-up, another farmer from the same neighborhood was chosen as a replacement.

In the initial farm visit, we recruited calves just days after birth, documenting their dates and histories. Each recruited calf, along with those born later, was assigned an ID. All selected calves received regular bimonthly visits until they turned 6 months old.

### 2.4. Sample Size Determination and Sampling Technique

Epi Info Statcalc 7.2.6.0 software was used to calculate the sample size for the prospective cohort study based on the following assumptions: a 95% confidence level, 80% power, a mortality rate of 0.0314 for normally born calves, a 0.21 mortality rate for calves born to cows with dystocia, and a hazard ratio (HR) of 9.3 from Abebe et al. [11]. The initial required sample size was 168, which was increased by 20% to 204 to account for potential dropouts. This final sample was distributed across three districts: 81 in Halaba, 62 in Misrak Badewacho, and 61 in Danboya. Additionally, 120 households from which the calves were recruited were included in the questionnaire survey, with 40 households selected from each district.

### 2.5. Data Collection

Data on calf health issues and potential risk factors were collected through follow-ups and interviews with owners or managers of smallholder dairy farms. The questionnaire aimed to gather information on management practices that may affect calf morbidity and mortality. Management factors were categorized into herd-level and calf-level aspects.

Farm-level factors encompassed descriptions of the farm, colostrum management, calf housing, health practices, breeding systems, and overall feeding strategies. Calf-level parameters included breed, birthplace, calving events, colostrum administration, initial housing, and observed health issues. Each calf was individually identified, and all incidents of illness and mortality were documented at each visit using a tailored data-recording format. Clinical examinations were conducted for any health issues to determine their causes, with standardized definitions for mortality, illness events, and treatments.

Mortality was defined as any calf death occurring more than 24 h after birth, regardless of the cause. Morbidity referred to observable clinical conditions in calves that could lead to death or require intervention. Calves were monitored until six months old, unless they were sold or otherwise lost, allowing for a maximum of 12 monitoring visits per calf. Additional emergency visits were conducted in response to health concerns reported by dairy farm owners. Dates and causes of loss were recorded for any calves lost during the follow-up, while check-off forms identified specific risk factors for each calf.

### 2.6. Statistical Analysis

Data from follow-ups and questionnaires were entered into Microsoft Excel, coded, and transferred to STATA version 14 (Stata Corp LLC, Texas, USA, 2017) for statistical analysis. Questionnaire results were summarized using descriptive statistics, including counts and percentages.

Morbidity and mortality events were assessed as true incidence rates, defined as the frequency of events per unit of animal time at risk [20]. All-cause or cause-specific incidence rates for morbidity or mortality were calculated by dividing the number of events during the observation period by the total animal time at risk. The periods at risk, expressed as calf-months, represent the total months calves were present in the study without experiencing disease or dying. Morbidity and mortality rates were reported per 100 calf-months at risk. A calf that recovered from one disease was considered at risk for contracting another when calculating the true morbidity rate.

The Kaplan–Meier (K-M) method estimated the survival function for calves, while a log-rank test (*p* < 0.05) evaluated differences in survival curves. K-M life table analysis was used to calculate the cumulative incidence or survival probability of morbidity and mortality. Multivariable Cox proportional hazard regression analysis was employed to identify the risk factors for calf morbidity and mortality, using “time to morbidity or mortality” as the outcome variable. The analysis included covariates such as the birth condition, calving process, birth timing and location, farm floor, sex, breed, naval disinfection, maternal colostrum feeding, colostrum ingestion timing and method, colostrum source, dam presence during feeding, calf vigor, separation time from the dam, weaning age, mothering instinct, dam's parity and breed, dam's health disorders, breeding method, and calf age. Potential variables were screened using the log-rank test, selecting factors with a *p* value < 0.2. The final multivariable Cox model for each outcome variable was constructed through stepwise backward exclusion of factors that were not statistically significant (*p* > 0.05). An 80% likelihood of accuracy was assumed to assess survival function equality, and confounding variables were identified as those causing a 20% change in the coefficients of other variables. The Schoenfeld and scaled Schoenfeld residual plots tested the proportional hazard assumption in the Cox model. Results were presented using HRs and 95% confidence intervals.

## 3. Results

### 3.1. Questionnaire Survey Result

A questionnaire survey conducted on 120 smallholder dairy farms identified common herd management practices. Of the respondents, 77% were from urban areas, and 23% were from periurban regions. Male-led farms made up 59.2%, while female-led farms accounted for 40.8%. In terms of education, 16.6% were illiterate, 54% completed primary school, 25% finished secondary education, and 4.2% attended college. For 34.2% of farmers, dairy production was the primary income source, while 65.8% considered it secondary. Most dairy farmers (77.5%) had over 5 years of experience, with 22.5% having less than 5 years.

Regarding breeding practices, 76.7% of farms used AI, resulting in 77.5% of calves born this way, while 23.3% practiced natural mating, accounting for 22.5% of calves. Most farms (78.3%) were managed by owners, with the remaining 21.7% managed by hired personnel. In terms of colostrum provision for calves, 65% of farms administered it immediately after birth and allowed the calves to remain with their mothers for extended periods. In contrast, 35% of farms provided colostrum only after 6 h and separated the calf from the dam after just 24 h. Concerning feeding methods for colostrum, 65.8% of farms opted for bucket-feeding, while 34.2% chose suckling. Only 35% of farmers provided naval treatment care to newborn calves. Approximately 42% provided free water access, while 58% allowed it intermittently. About 39.2% of farms practiced free grazing, 50% stall feeding, and 10.8% partial grazing after weaning. Indoor calving was practiced by 80.8% of farmers, whereas 19.2% calved outdoors. Additionally, 38.3% used cow sheds for calves, while 61.7% had separate pens, and 88.3% provided bedding in calf houses, leaving 11.7% without. The majority of farms (97.5%) clean calf pens daily.

### 3.2. Calf Morbidity and Mortality Rates

In this study involving 204 monitored calves, 101 (49.5%) showed signs of morbidity, and 31 (15.2%) died from various causes. The calves contributed a total of 756.5 calf months at risk for morbidity and 767.8 calf months at risk for mortality. The median duration of morbidity was 4.9 months, while median survival for mortality was 1.3 months. The overall morbidity and mortality rates were calculated at 13.4 (95% CI: 11.3–15.2) and 4.0 (95% CI: 2.8–5.4) cases per 100 calf months at risk, respectively (Table 1).

### 3.3. Morbidity and Mortality Causes

Diarrhea is the primary cause of illness and death, contributing to 50.5% of morbidity and 64.5% of mortality. Septicemia ranks as the second leading cause of morbidity at 14.9%, while pneumonia is the second leading cause of mortality at 19.4%. Tables 2 and 3 below list additional common causes of morbidity and mortality identified in the study. The incidence rates for diarrhea, septicemia, and pneumonia are 7.8, 2.1, and 1.9 cases per 100 calf-months at risk, respectively (Table 2). The mortality rates for these conditions are 2.7, 0.8, and 0.5 cases per 100 calf-months, respectively (Table 3).

### 3.4. Risk Factors for Calf Morbidity and Mortality

#### 3.4.1. K-M Survival Analysis

**Figure 2:**
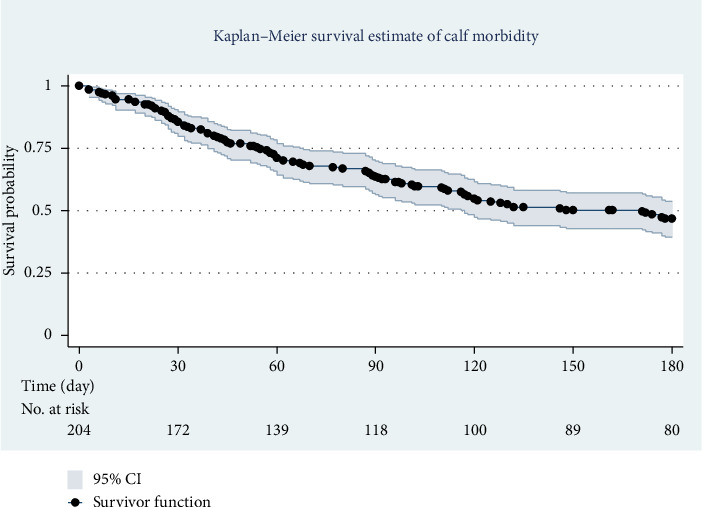
K-M survival curve of all-cause morbidity in calves from birth to 180 days of age.

**Figure 3:**
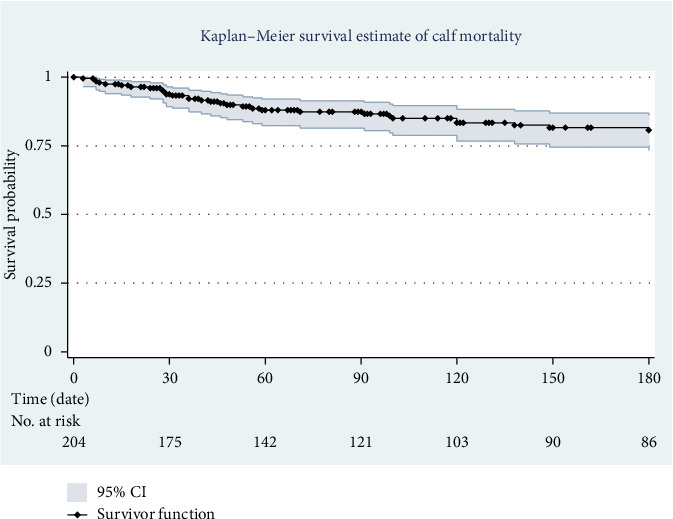
K-M survival curve of all-cause mortality in calves from birth to 180 days of age.

K-M survival analysis was employed in this study to assess the time until disease occurrence or death, particularly useful for censored data where not all participants experienced the event by the study's end. This method allowed us to estimate survival probabilities, identify risk factors for morbidity and mortality, analyze time-to-event data, and compare survival curves across groups. The K-M survival curve for calf morbidity shows a decreasing probability of survival with age, indicating an increased likelihood of disease as calves grow older (Figure 2). Similarly, the K-M mortality curve reveals a slight decline in calf survival probability with age (Figure 3). Additionally, we conducted a K-M life table analysis to assess survival probability for morbidity and mortality from birth to 6 months. The survival rates for calves during this period were 46% for morbidity and 79% for mortality. During the follow-up, 13 calves were sold, while 90 remained healthy and were monitored until the study's conclusion (Table 4).

#### 3.4.2. Univariable Analysis (Log-Rank Test)

The study used a log-rank test to compare survival curves and identify predictors for multivariable Cox proportional hazard regression analysis. Ten variables were significantly associated with the morbidity risk of calves (*p* < 0.05), including the calving condition, site of birth, calf house flooring, sex, time of colostrum ingestion, mothering instinct during feeding, weaning age, dam's breed and health disorder, and calf age. These variables were selected for multivariable analysis. Additionally, factors significantly associated with calf mortality risk (*p* < 0.05) in the log-rank test included the calving condition, time and site of birth, calf house flooring, weaning age, calf sex, dam's breed, source of colostrum, and calf age (Table 5).

#### 3.4.3. Multivariate Cox Regression Analysis

##### 3.4.3.1. Calf Morbidity

In the multivariable Cox regression analysis, seven factors significantly (*p* < 0.05) associated with morbidity risk were identified: calving assistance, calf house floor condition, calf sex, timing of colostrum intake, weaning age, dam breed, and calf age. After adjusting for other variables, calves born to assisted dams had a 1.93 times higher morbidity risk than those born normally. Calves from farms with nonconcrete floors faced a 2.88 times higher risk compared to those from concrete-floored farms. Male calves had a 1.86-fold increased risk of morbidity compared to females. Calves that ingested colostrum late after 6 h of birth had 1.7 times higher morbidity risk than those that received colostrum immediately after birth. Additionally, morbidity risk decreased by 53%, 79%, and 73% for calves weaned after 90 days, born to local breed dams, and older than 90 days, respectively, when controlling for other factors (Table 6). The final model met the proportional hazard assumption (global test: Chisq. = 12.53; df = 7; *p*=0.84).

##### 3.4.3.2. Calf Mortality

The multivariate Cox proportional hazard analysis identified seven independent predictors of calf mortality: farm location, calving assistance, site of birth, type of flooring on the farm, timing of colostrum intake, weaning age, and calf age. Calves born to dams that required assistance had a 7.7-fold higher risk of death compared to those born unassisted. Calves delivered outdoors faced a 27.3-fold increase in mortality risk compared to those born indoors. Births that occurred on nonconcrete floors were associated with a 9.2-fold greater risk of mortality relative to those on concrete surfaces. Calves that received colostrum later than 6 hours after birth had a 7.7-fold increased risk of death compared to those fed immediately after birth. In contrast, calves raised in urban settings experienced a 75% reduction in mortality risk. Additionally, calves fed with their dam present during bucket feeding showed a 95% reduction in risk, while calves weaned after 90 days had a 97% lower risk. Calves older than 90 days also experienced an 85% reduction in mortality risk. All results were obtained after adjusting for other variables (Table 7). The final model satisfied the proportional hazard assumption, with a global test result of Chi-square = 17.4; degrees of freedom = 9; and *p*=0.19.

## 4. Discussion

This investigation tracked 204 newborn calves over six months, observing a morbidity percentage of 49.5% and a mortality percentage of 15.2% from various causes. The mortality figures are alarming, surpassing the acceptable threshold of 3% to 5% for well-managed calves [15, 21]. Numerous studies in Ethiopia and globally have reported calf morbidity and mortality rates using inconsistent methods and definitions of “rate.” According to precise definitions, “rate” should reflect measurements based on animal-time units, specifically as a ratio where the denominator is the total animal-time units at risk [20]. Our study estimates adhere to this definition, yielding an all-cause morbidity rate of 13.4 cases per 100 calf months and a mortality rate of 4.04 cases per 100 calf months at risk. Our findings align with those of Hordofa et al. [10] and Abebe et al. [11], who reported rates of 13.81 and 4.12 cases and 12.7 and 3.7 cases per 100 calf months at risk, respectively. In contrast, Alemu et al. [22] reported higher rates of 64 and 19 cases per 100 calf months at risk. Similarly, Ahmedin and Assen [12] found a morbidity rate of 55 and a mortality rate of 14 per 100 calves over six months. Variations in findings may result from differences in sample sizes, population characteristics, and geographic conditions [23].

The study found that calves needing assistance during calving have a significantly higher risk of morbidity (HR = 1.9) and mortality (HR = 7.7) than those born without assistance. These results are consistent with previous research by Hordofa et al. [10], Abebe et al. [11], and Ahmedin and Assen [12]. Calves from difficult births are more susceptible to infectious diseases such as diarrhea and respiratory illnesses due to stress and potential injuries during prolonged or complicated deliveries. Extended labor can lead to hypoxia, resulting in weak calves that struggle to suckle and absorb colostral antibodies, increasing their risk of illness and death. Difficult births can also hinder the passive transfer of immunoglobulins from the dam, compromising the calf's immune system. Furthermore, the impacts of dystocia can extend beyond the immediate postnatal period, with surviving calves potentially facing long-term health issues that affect their growth and productivity [24, 25]. This highlights the need for effective management and timely intervention during calving to reduce these risks and improve calf survival rates.

In this study, calves raised on nonconcrete floors had a higher risk of morbidity (HR = 2.9) and mortality (HR = 9.2) compared to those on concrete floors. This aligns with Ahmedin and Assen [12], who found that calves on mud floors were at greater risk of death than those on concrete. Concrete flooring provides a stable, easy-to-clean surface that reduces bacterial growth, while mud floors are challenging to maintain dry and clean [14]. Although mud flooring is gentler on joints, it poses hygiene challenges, potentially elevating the risk of microbial infections compared to calves raised on concrete [26, 27].

Our findings demonstrate that calves receiving their first colostrum intake after more than 6 hours postbirth had a significantly higher risk of both illness (HR [HR = 1.7]) and mortality (HR = 7.68), compared to those fed immediately after birth. This aligns with previous research, including Tora et al. [28] and Alemu et al. [22], and echoes clinical trials, and observational studies showing delayed colostrum feeding markedly increase mortality risk—one study reported a fivefold increase in mortality hazards among late-fed calves [29, 30]. The elevated morbidity and mortality late-fed calves are likely due to the inadequate passive transfer of immunity [31, 32]. Newborn calves are agammaglobulinemic at birth and rely entirely on colostrum-derived immunoglobulin G (IgG) for systemic immunity [29]. Timely colostrum delivery is essential because immunoglobulin absorption sharply declines after gut closure, which begins within hours of birth. Delays not only reduce serum IgG levels but also increase susceptibility to infections during the preweaning period [31, 33]. Therefore, these findings reinforce the critical importance of timely colostrum feeding—ideally within the first 2 h of life, supplying both adequate volume and high quality—to maximize IgG transfer, enhance neonatal immune protection, and reduce calf morbidity and mortality.

Based on the current study, it was found that male calves had a significantly higher likelihood of falling ill compared to females (HR = 1.86), although there was no notable difference in mortality risk between the two genders. This elevated risk of illness among male calves has also been observed in previous studies [11, 34], which also reported a higher incidence rate of morbidity in male calves compared to females. Abebe et al. [11] suggested that male calves generally receive less care in terms of feeding, medical attention, and overall management, making them more susceptible to infections. On the contrary, female calves are considered more valuable for future breeding and are regarded as being economically more important. Consequently, they receive better care and attention from dairy farmers.

Calves weaned at or after 90 days exhibited a 53% lower risk of illness and a 97% reduced risk of mortality compared to those weaned earlier. This is consistent with Tora et al. [28] and Abebe et al. [11], which found higher mortality rates among calves weaned before 3 months. It is known that weaning at or after 90 days allows for better immune and digestive system development. In contrast, early weaning can stress calves that are still maturing physically leading to difficulties with dietary and social adjustments [35, 36].

In comparison to calves born to cross-breed dams, calves born from local-breed dams exhibited a 79% decrease in the risk of morbidity. This aligns with the findings of Tora et al. [28], which indicated that crossbred calves had a higher risk of morbidity than local breed calves. The lower morbidity risk in local breed calves might be attributed to factors such as environment adaptation, disease resistance, nutritional efficiency, maternal care, and genetic diversity inherent in local cattle breeds compared to crossbreeds [37, 38]. These findings underscore the critical role that local cattle breeds play in safeguarding livestock health and enhancing agricultural sustainability. Efforts to promote the conservation and utilization of these breeds are essential not only for maintaining genetic diversity but also for ensuring the resilience of farming systems in the face of changing climates and emerging diseases.

The study revealed that calves aged three or older had a 77% lower risk of getting sick and an 85% lower risk of dying compared to calves under 3 months. It was observed that most of the morbidities (71.3%) and mortalities (71%) occurred in calves under 3 months. This agrees with previous studies by Wudu et al. [17], Ferede et al. [39], and Abebe et al. [11]. Ahmedin and Assen [12] also pointed out that the first 4 months of a calf's life present the highest risk of morbidity and mortality. These findings highlight how important it is to take good care of calves during their early months, as early mortality impacts future cow populations and genetic progress.

Calves born outdoors had a 27.3-fold higher risk of mortality than those born indoors. This elevated risk, as observed during the study and supported by Fentie et al. [40], can be attributed to adverse weather exposure, disease risk, insufficient oversight, challenges in ensuring adequate colostrum intake, and poor biosecurity. Therefore, effective colostrum management is crucial for the health and survival of outdoor-born calves, particularly ensuring that they receive colostrum from their dam [24].

Diarrhea was identified as the primary cause of calf morbidity and mortality in this study, followed by pneumonia as the second leading cause of mortality and septicemia as the second most common cause of morbidity. Consistent to the present study, diarrhea and pneumonia have been consistently documented as the two most common causes of calf morbidity and mortality in various regions of the Ethiopia [10–12, 22, 28, 41]. According to studies, factors contributing to the occurrence of diarrhea in calves include poor hygiene, inadequate management practices, insufficient veterinary care, low colostrum intake, and high levels of stress, which diminish passive immunity and increase young calves' susceptibility to diarrhea-causing pathogens [10, 11, 41, 42]. Diarrhea, often caused by infectious agents such as *Escherichia coli* and rotavirus, leads to dehydration and nutrient mal-absorption, compromising the overall health and growth of calves [43, 44].

The median survival times for calves with diarrhea, pneumonia, and septicemia were 52, 32, and 67 days, respectively. These results highlight the vulnerability of neonatal calves under 90 days old to high morbidity from these conditions, underscoring the urgent need for targeted interventions and management strategies during this critical period. Due to their immature immune systems, neonatal calves require effective preventive measures, including proper nutrition, vaccination, and biosecurity practices. Early identification of morbidity signs can significantly improve survival rates [45, 46]. Pneumonia, often caused by pathogens such as Mannheimia haemolytica and Pasteurella multocida, can lead to severe respiratory distress and death if untreated, adversely affecting calf welfare and productivity [47].

## 5. Limitation of the Study

A major limitation of this study was its reliance on visible calf health disorders and mortalities without the use of diagnostic tests or lab procedures due to financial constraints. Additionally, no samples from affected or deceased calves were collected for laboratory testing, making it difficult to identify specific causative agents for the health issues. Future studies should prioritize the use of advanced diagnostic techniques to determine the specific causes of calf morbidity and mortality.

## 6. Conclusion

The study found calf morbidity and mortality rates comparable to previous research in the country, but the mortality percentage exceeded the economically acceptable level for well-managed farms. This high calf morbidity and mortality in smallholder dairy farms adversely affects the availability of healthy calves, which is crucial for sustaining the sector and improving farmers' livelihoods. The research identified various disease syndromes affecting calf health, with diarrhea being the most prevalent. Significant risk factors linked to morbidity and mortality included farm location, calving assistance, birth site, farm floor, timing of colostrum intake, weaning age, and calf age. Thus, effective colostrum management—ensuring newborns receive adequate dam colostrum promptly—and early disease detection and treatment are vital to reducing calf morbidity and mortality. It is also important to educate dairy farmers on best management practices to lower these rates in their herds. Future studies should prioritize identifying specific causes of calf morbidity and mortality to implement targeted control measures.

## Figures and Tables

**Table 1:** Morbidity and mortality rates in calves under 6 months of age based on sex, farm location, and district.

Variable		Calf at risk	Cases	Time at risk (month)	IR/100 calf month	95% CI for IR
*Morbidity*						
Sex	Female	121	50	474.1	10.54	8.4–13.3
	Male	83	51	282.4	18.0	16.7–22.4
Farm location	Urban	144	75	551.2	13.6	11.8–16.2
	Periurban	60	26	205.3	12.6	9.3–14.2
Districts	Halaba	81	67	224.9	29.8	22.8–34.6
	Danboya	61	12	263.7	4.5	3.6–5.7
	Misrak Badewacho	62	22	267.8	8.2	7.1–10.4
Total		204	101	756.5	13.4	11.3–15.2

*Mortality*						
Sex	Female	121	17	479.8	3.5	2.2–4.8
	Male	83	14	287.9	4.8	3.2–6.7
Farm location	Urban	60	8	208.0	3.8	2.6–5.5
	Periurban	144	23	559.8	4.1	2.9–5.9
Districts	Halaba	81	20	231.2	8.6	6.7–9.2
	Danboya	61	5	265.7	1.8	1.2–1.5
	Misrak Badewacho	62	6	270.8	2.2	1.5–3.6
Total		204	31	767.8	4.04	2.8–5.4

Abbreviations: CI = confidence interval, IR = incidence rate.

**Table 2:** Causes of calf morbidity and their incidence rate.

Disease condition	Number of cases	Percentage (%)	Calf month at risk	Incidence rate/100 calf month
Diarrhea	51	50.5	654.8	7.8
Pneumonia	14	13.9	728.2	1.9
Septicemia	15	14.9	717.2	2.1
Navel infection	6	5.9	745.1	0.8
Eye problem	3	3	745.4	0.4
Unknown	12	11.9	722.3	1.7

All causes	101	100	756.5	13.4

**Table 3:** Causes of calf mortality and their incidence rate.

Disease condition	Number of cases	Percentage (%)	Calf month at risk	Incidence rate/100 calf month
Diarrhea	20	64.5	730.3	2.7
Pneumonia	6	19.4	759.7	0.8
Septicemia	4	12.9	759.3	0.5
Unknown	1	3.2	764.5	0.1

All causes	31	100	767.8	4.04

**Table 4:** Age-specific cumulative survival and incidence of all-cause morbidity and mortality in calves under 180 days old.

Age interval (days)	No. at risk	Cases	Censored	Proportion surviving (%)	Cum incidence (%)	95% CI
*Morbidity*						
0–30	204	27	5	86	13	9–19
30–60	172	27	6	73	27	21–34
60–90	139	16	5	64	36	29–43
90–120	118	15	3	56	44	37–51
120–150	100	10	1	50	50	43–57
150–180	89	6	3	46	54	46–60
180-	80	0	80	46	54	46–60

*Mortality*						
0–30	204	12	17	94	6	3–10
30–60	175	10	23	88	12	8–17
60–90	142	1	20	87	12	8–18
90–120	121	3	15	85	15	10–21
120–150	103	4	9	82	18	13–25
150–180	90	0	4	82	18	13–25
180-	86	1	85	79	21	14–28

Abbreviation: CI = confidence interval.

**Table 5:** Log-rank test of predictors of morbidity and mortality in calves for survival function equality.

No.	Predictors	Category	Morbidity		Mortality	
			Chisq.	*P*	Chisq.	*P*
1	Dairy farm location	Urban vs. periurban	0.26	0.600	0.07	0.001
2	Calving condition	Assisted vs. not	9.36	0.009	10.7	0.004
3	Time of birth	Day vs. night	12.8	0.087	15.8	< 0.001
4	Site of birth	Outdoor vs. indoor	4.41	< 0.001	7.86	0.005
5	Floor structure	Nonconcrete vs. concrete	27.6	0.002	8.92	0.002
6	Sex	Male vs. female	6.71	0.018	3.91	0.047
7	Calf breed	Cross vs. local	0.02	0.494	0.14	0.710
8	Navel disinfection	Yes vs. no	2.6	0.131	0.00	0.960
9	Time of colostrum ingestion	≤ 6 h vs. > 6 h	15.3	0.001	2.65	0.440
10	Source of colostrum	Dam vs. another cow	3.40	0.224	6.52	0.038
11	Vigor status	Good vs. poor	0.12	0.092	0.26	0.611
12	Weaning age	< 90 days vs. ≥ 90 days	16.96	< 0.001	50.77	< 0.001
13	Mothering instinct	Good vs. poor	0.75	0.007	0.35	0.556
14	Parity of the dam	Primiparous vs. multifarious	2.57	0.109	0.09	0.763
15	Dam breed	Cross vs. local	11.55	< 0.001	5.01	0.025
16	Dam health disorders	Yes vs. no	7.75	0.005	1.19	0.275
17	Age of calf	< 90 days vs. ≥ 90 days	25.81	< 0.001	6.76	0.009

**Table 6:** Multivariate Cox proportional hazard regression analysis of explanatory variables associated with calf morbidity.

Variables	Category	HR	95% CI	*p* value
Calving	Not assisted	Ref		
	Assisted	1.93	1.46–2.56	< 0.001

Floor of the farm	Concrete	Ref		
	Nonconcrete/mud	2.88	1.67–4.96	< 0.001

Sex	Female	Ref		
	Male	1.86	1.14–3.02	0.012

Timing of colostrum intake	Immediately	Ref		
	> 6 h	1.7	1.1–2.6	0.018

Calf weaning age	< 90 days	Ref		
	≥ 90	0.47	0.29–0.74	0.001

Dam breed	Cross	Ref		
	Local	0.21	0.11–0.42	< 0.001

Age	< 90 days	Ref		
	≥ 90 days	0.23	0.14–0.37	< 0.001

Abbreviations: CI = confidence interval, HR = hazard ratio.

**Table 7:** Multivariate Cox proportional hazard regression analysis of explanatory variables associated with calf mortality.

Variables	Category	HR	95% CI	*p* value
Farm location	Periurban	Ref		
	Urban	0.25	0.07–0.88	0.032

Calving assistance	Not assisted	Ref		
	Assisted	7.70	2.94–20.2	< 0.001

Site of birth	Indoor	Ref		
	Outdoor	27.3	5.73–130	< 0.001

Floor structure	Concrete	Ref		
	Nonconcrete/mud	9.18	2.79–30.2	< 0.001

Timing of colostrum intake	Immediately	Ref		
	> 6 h	7.68	2.09–28.1	0.002

Weaning age	< 90 days	Ref		
	≥ 90 days	0.03	0.00–0.16	< 0.001

Calf age	< 90 days	Ref		
	≥ 90 days	0.15	0.04–0.50	< 0.001

Abbreviations: CI = confidence interval, HR = hazard ratio.

## Data Availability

The data that support the findings of this study are available from the corresponding author upon reasonable request.
